# Supplementary material for: Diet, Sun, Physical Activity and Vitamin D Status in Children with Inflammatory Bowel Disease
Source: Nutrients. 2022 Feb 28;14(5):1029. doi: 10.3390/nu14051029 (PMC8912613; doi:10.3390/nu14051029)
Supplement: Supplementary file 1 [file nutrients-14-01029-s001.zip › nutrients-1613046-supplementary.pdf]

nutrients MDPI

Supplementary tables:

Table S1. Serum vitamin D level in study group depending on the clinical course of the disease, past medical history, risk factors and medications intake (Y-yes, N-no); mean+/- SD, median (1st-3rd quartile); ANOVA, Kruskal-Wallis One Way Analysis of Variance on Ranks.

Characteristic	25OHD [ng/ml] (mean+/-SD / median (25%-75%)				
	**IBD**	**CD**	**UC**	**C**	**P**
PCDAI/PUCAI:					
1		19.75 (13.7-22.0)	18.35±6.82		
2		17.6 (14.3-23.55)	19.54±11.49		
3		25.9 (20.25-41.5)	13.47±1.58		NS
4		14.65 (6.1-27.55)			
Location:					
L1/E1		16.3 (14.5-18.1)	12.96±6.85		
L2/E2		19.5 (14.2-25.6)	19.66±8.66		NS
L3/E3		22 (18.4-24.57)	19.12±10.42		
L4/E4		-	18.92±8.76		
Fractures	Y – 19.8 (13.55-26.63)<br>N – 18.1 (14.2-22.68)	*	*	Y – 16.9 (15.85-18.25)<br>N – 14.85 (11.55-19.15)	NS
Surgical abdominal intervention	**Y – 22.0 (19.0-23.28)**<br>**N – 17.3 (13.65-23.43)**	*	*		NS

*due to a small sample size, calculations were made only for IBD/C group.

Table S2 Anthropometric values of IBD patients in two subgroups – those with vitamin D deficiency and with D level normal ; t-test.

	**<20 ng/ml**	**>20 ng/ml**	**P**
Weight z-score	-0.76±1.03	-1.04±1.06	0.305
Height z-score	-0.36±1.22	-0.69±1.34	0.326
BMI z-score	-0.77±0.86	-0.77±0.86	0.6

Table S3. Vitamin D level and BMI z-score in IBD patients; ANOVA, p=0.29.

BMI z-score	Number of patients	Vit D level (ng/ml)
< -2	8	18.1 (11.65-20.6)
-2 to -1	13	19.3 (14.42-26.28)
-1 to 1	38	17.9 (14.2-23.3)
1 to 2	3	13.5 (4.12-18.15)
> 2	-	-

Table S4. Frequency of physical activity of IBD and control group in different times of the year (1=everyday, 2=few times per week, 3=few times per month, 4=less than 1x per month).

Season	Subgroup	Number of patients in %					
		1	2	3	4	1+2	P
**Spring**	CD	41.18	44.12	5.88	8.82	85.30	0.12
	UC	25.0	57.14	10.71	7.14	82.14	
	C	44.68	53.19	2.13	0.00	97.87	
Summer	CD	47.06	35.29	8.82	8.82	82.35	0.39
	UC	50.00	32.14	10.71	7.14	82.14	
	C	48.94	38.30	12.77	0.00	87.24	
Autumn	CD	35.29	47.06	11.76	5.88	82.35	0.09
	UC	25.00	57.14	14.29	3.57	82.14	
	C	40.43	57.45	2.13	0.00	97.88	
Winter	CD	32.35	44.12	11.76	11.76	76.47	0.17
	UC	17.86	60.71	14.29	7.14	78.57	
	C	31.91	61.70	6.38	0.00	93.61	

Table S5. Frequency of physical activity throughout the year and vitamin D level in total group of patients.

Points	Vitamin D level
0	12.95 (11.2-14.7)
2	12.1 (12.1-12.1)
4	14.5 (13.97-17.67)
5	19.3 (19.3-19.3)
6	19.15 (15.7-22.9)
7	13.8 (7.45-25.0)
8	17.2 (13.4-23.62)
9	18.7 (12.87-22.45)
10	17.6 (12.5-22.55)
11	13.95 (10.15-14.65)
12	17.5 (14.2-21.0)
